# Short-term isolation effects on the brain, cognitive performance, and sleep—The role of exercise

**DOI:** 10.3389/fphys.2023.903072

**Published:** 2023-01-30

**Authors:** Timo Klein, Leonard Braunsmann, Jessica Koschate, Uwe Hoffmann, Tina Foitschik, Stephanie Krieger, Brian Crucian, Stefan Schneider, Vera Abeln

**Affiliations:** ^1^ Institute of Movement and Neuroscience, German Sport University Cologne, Cologne, Germany; ^2^ University of Rostock, Institute of Sport Science, Rostock, Germany; ^3^ Centre for Health and Integrative Physiology in Space (CHIPS), German Sport University Cologne, Cologne, Germany; ^4^ VasoActive Research Group, School of Health and Sport Sciences, University of the Sunshine Coast, Maroochydore, QLD, Australia; ^5^ Geriatric Medicine, Department for Health Services Research, School of Medicine and Health Sciences, University of Oldenburg, Cologne, Germany; ^6^ KBR, Houston, TX, United States; ^7^ NASA-Johnson Space Center, Houston, TX, United States; ^8^ Institute of Movement and Neurosciences, Center for Health and Integrative Physiology in Space, German Sport University Cologne, Cologne, Germany; ^9^ School of Maritime Studies, Memorial University of Newfoundland, St. Johns, NL, Canada; ^10^ Faculty for Science, Health, Education and Engineering, University of the Sunshine Coast, Maroochydore, QLD, Australia

**Keywords:** confinement, mental health, cortisol, stress, neurotrophic factors, physical activity, cognition, space flight

## Abstract

Isolation is stressful and negatively affects sleep and mood and might also affect the structure and function of the brain. Physical exercise improves brain function. We investigated the influence of physical exercise during isolation on sleep, affect, and neurobehavioral function. N = 16 were isolated for 30 days with daily exercise routines (ISO_100_) and *n* = 16 isolated for 45 days with every second day exercise (ISO_50_). N = 27 were non-isolated controls who either exercised on a daily basis (CTRL_Ex_) or refused exercise (CTRL_NonEx_) for 30 days. At the beginning and the end of each intervention, intravenous morning cortisol, melatonin, brain-derived neurotrophic factor and IGF-1, positive and negative affect scales, electroencephalography, cognitive function, and sleep patterns (actigraphy) were assessed. High levels of cortisol were observed for the isolated groups (*p* < .05) without negative effects on the brain, cognitive function, sleep, and mood after 4 to 6 weeks of isolation, where physical exercise was performed regularly. An increase in cortisol and impairments of sleep quality, mood, cognitive function, and neurotrophic factors (*p* < .05) were observed after 4 weeks of absence of physical exercise in the CTRL_NonEx_ group. These findings raise the assumption that regular physical exercise routines are a key component during isolation to maintain brain health and function.

## 1 Introduction

Life in an isolated and confined environment is challenging for astronauts during space travel. Recently, isolation has also affected people on Earth during lockdown periods of the COVID-19 pandemic. The primary question of this investigation is if life in an isolated and confined environment for a short duration of time is harmful for the brain and cognitive function.

The effect of isolation on cognitive performance shows disparities. The majority of research reported impairments in cognitive function, including problem-solving tasks (Sauer et al., 1999), working memory (Reed et al., 2001), executive function (Cacioppo et al., 2000), and attentional performance (Basner et al., 2013), whereas some showed a maintained cognitive performance (Abeln et al., 2015; Barkaszi et al., 2016). These studies used isolation periods of up to 520 days. While impairments in cognitive function have been controversially shown in long-term isolation studies, current research is missing on short-term isolation effects. There is also a need to understand the underlying mechanisms and timing of these impairments in cognitive functions to adequately design countermeasures and overcome potential negative effects of isolation on cognitive performance.

Cognition and affect are strongly linked and may provide some understanding for impairments observed in cognitive performance during isolation. The state of affect refers to the underlying experience of feeling, emotion, or mood (Hogg et al., 2010). It is well-established that mood deteriorates during long-term social isolation (De La Torre et al., 2012). Social isolation showed impaired self-regulation of hedonistic processes (Baumeister et al., 2005), which is the pursuit of pleasure and positive emotions under the absence of negative emotions (Kringelbach and Berridge, 2010). Positive emotions are a key psychological component as they enhance the ability to cope with stressful events (Tugade and Fredrickson, 2004). The state of affect influences the interaction of an individual with stimuli including the cognitive scope and thus cognitive performance. Thus, preserving the state of affect is of high importance for periods of isolation.

Another potential explanation for the impairment in cognitive performance during longer periods of isolation may be a disrupted stress hormonal regulation *via* an overstimulation of the hypothalamic–pituitary–adrenal axis (HPA axis) (Choukèr et al., 2002; Jacubowski et al., 2015). In addition to increased hormonal stress, there is also psychosocial stress, which has adverse effects on neurotrophic factors and hence cognitive performance. Growing evidence shows that psychosocial stress can cause damage and atrophy in certain brain areas, such as the hippocampus and the prefrontal cortex (Duman and Monteggia, 2006; Liston et al., 2009). This is assumed to be linked to reduction of brain-derived neurotrophic factor (BDNF) in these areas, an important regulator for neurogenesis and neuroplasticity. Reduction in BDNF levels have also been shown during social isolation (Barrientos et al., 2003; Gong et al., 2017). Insulin-like growth factor 1 (IGF-1), known for its positive influence on cell proliferation and neurogenesis in the adult brain (Anderson et al., 2002), may be negatively affected by psychosocial stress due to a reduced expression of IGF-1 *via* inhibiting the effects of upregulated glucocorticoid levels (Anderson et al., 2002). The investigation of neurotrophic factor regulation together with cognitive performance during isolation helps in examining an important underlying mechanism.

Isolation also affects the brain in its cortical activation. Long-term isolation reduces cortical activity on a global level (Schneider et al., 2010), where reduced neuronal activation has recently been proposed as a neuronal adaptive mechanism of the brain as a response to potential sensory deprivation during long-term isolation (Weber et al., 2020). It is important to investigate cortical activation changes alongside cognitive performance to provide a better understanding of the underlying mechanisms within the brain and their significant contribution in emotional processing and integrating different sensory modalities to form multiple cognitive functions (Andersen, 1997; Brodt et al., 2016).

High levels of stress during isolation may impair sleep quality and directly impair cognitive function. Recent evidence in humans suggested that even perceived social isolation leads to poor sleep outcomes (Cacioppo et al., 2000). Poor sleep was consistently observed during long-term isolation (Brown et al., 2017; Mairesse et al., 2019; Zivi et al., 2020) and is one of the adverse outcomes of social isolation common to humans (Cacioppo et al., 2000). Impairments in sleep quality and quantity during long-term isolation (Mairesse et al., 2019) might negatively affect the central nervous system function, neurotrophic factors, and brain cortical activation (Krause et al., 2017) simultaneously.

Physical exercise is known for its positive effects on the brain, and a useful strategy is proposed to overcome the negative effects of isolation. It is well-accepted that physical exercise promotes neurogenesis due to upregulation of neurotrophic factors (Miranda et al., 2019), improves brain cortical activation (Schneider et al., 2010) and mood (Abeln et al., 2015), and positively influences sleep (Kline et al., 2021) and hence cognitive performance (Gomez-Pinilla and Hillman, 2013; Mandolesi et al., 2018). Recent investigations focusing on space travel investigated the effect of isolation and confinement only on single factors such as mood (Schneider et al., 2013), cognition (Basner et al., 2014) and brain cortical activation (Abeln et al., 2015), sleep (Zivi et al., 2020), and exercise (Schneider et al., 2013; Abeln et al., 2015). The aim of this study was to investigate the effect of exercise during isolation and potential underlying factors including state of affect, stress, neurotrophic factors, brain cortical activation, and sleep altogether to provide a comprehensive picture and support the preservation of neuro-psychological function (cognitive performance) during isolation. We hypothesized that physical exercise during isolation would maintain the state of affect, neurotrophic factors, brain cortical activation, and sleep.

## 2 Materials and methods

### 2.1 Participants

This study included four groups: two isolated experimental groups (ISO) and two non-isolated control groups (CTRL). In total, 32 adults participated in the isolation groups and 27 adults participated in the control groups (Table 1). Both isolation groups were selected by the Human Exploration Research Analog (HERA) project after an open call following astronaut selection criteria as per current guidelines, which also provides a brief description and visualization of the isolation conditions (Cromwell and Neigut, 2014). The studies were part of the HERA programs campaign 3 (C3) and campaign 4 (C4), executed by the National Aeronautics and Space Administration (NASA, Houston, United States) and participation of the German Space Agency (DLR). The non-isolated control groups were selected based on the same criteria at the German Sport University Cologne, Germany. All experimental procedures conformed to the Declaration of Helsinki and were approved by the Human Research Multilateral Review Board of NASA (ID: Pro 1907) and the Ethics Committee of the German Sport University Cologne (Protocol Number 80/2015). A detailed verbal and written explanation of the study was provided, and written informed consent was obtained from each participant before participation.

**TABLE 1:** Participants’ characteristics.

	*IS O*_*100*_ *(n = 16)*	*IS O*_*50*_ *(n = 16)*	*CTRL*_*Ex*_ *(n = 17)*	*CTRL*_*NonEx*_ *(n = 10)*	*F (df)*	*n*_*p*_^*2*^	*p*
Male: female	9:7	16:6	8:9	7:3			
Age [years]	36 (32–40)	40 (36–44)	32† (28–36)	26*† (21–31)	6.87 (3,55)	.27	<.01
Height [cm]	173 (168–178)	175 (170–180)	176 (171–180)	178 (171–184)	.63 (3,55)	.03	.60
Weight [kg]	74 (66–81)	75 (67–83)	73 (66–80)	72 (62–83)	.08 (3,55)	<.01	.99
BMI [kg/m2]	24.6 (22.8–26.4)	24.0 (22.3–25.8)	23.3 (21.6–25.0)	22.7 (20.5–24.9)	.74 (3,55)	.04	.53

Values are displayed as mean ± SD. ISO_100_: isolated group 100% exercise; ISO_50_: isolated group 50% exercise; CTRL_Ex_: non-isolated exercise control group; CTRL_NonEx_: non-isolated non-exercise control group; BMI: body mass index. * Difference to ISO_50_ (*p* < .05); † difference to ISO_100_ (*p* < .05).

### 2.2 Study overview

The two isolation groups were isolated in two campaigns for either 30 days (HERA C3) or 45 days (HERA C4) with four missions of four participants. Sleep in HERA C4 was restricted to 5 h per night from Mondays to Fridays and 8 h on Saturdays and Sundays. During HERA C3, participants exercised for 30 days on a daily basis for 30 min 5 days per week and rested on weekends (ISO_100_). During HERA C4, participants exercised for 45 days in isolation every second day for 30 min (ISO_50_). Exercise routines alternated between bicycling and resistance exercise at a self-chosen moderate intensity and were restricted to not exceed 85% of the maximum age-adjusted heart rate during bicycling for safety reasons. The non-isolated control groups were separated into an exercise-control group (CTRL_Ex_) and a non-exercise control group (CTRL_NonEx_). The CTRL_Ex_ followed the same daily exercise protocol and had similar exercise habits as those of ISO_100_ for 30 days. The CTRL_NonEx_ were active individuals, habitually exercising 3 times per week for the past 3 years, but were not allowed to do any exercise for 30 days during the investigation period. Also, the commute by bicycle to work was considered an exercise and was restricted. In the CTRL_NonEx_ only, a cardiorespiratory fitness test until maximal voluntary exhaustion was performed prior to and after the intervention.

Data were collected prior to, after, and during the intervention in the ISO_100_, CTRL_Ex_ and CTRL_NonEx_ groups on days 7, 14, and 28 and in the ISO_50_ group at days 3, 10, 24, and 38. For the purpose of this study, the first time-point during the intervention (ISO and CTRL) was defined as T1 and the last time-point during each intervention (ISO and CTRL) as T2 and used for data comparison. Retrospectively, pre-isolation measurements (data collection 5 days prior to intervention) may not be ideal as baseline measurements in the isolated groups, where these periods are busy due to numerous tests and introductions prior to the start of the space-analog missions and possibly influence the results. Pre- and post-intervention results were reported separately (Supplementary Table S1). The assessments included an intravenous blood collection in the morning, followed by a sleep questionnaire and a sleep diary. In the course of the day, resting electroencephalography (EEG), PANAS-X questionnaire, and cognitive test batteries were collected, which are described in detail in the Experimental measures section. Parts of the results of the ISO_100_ and the CTRL_Ex_ groups were published previously (Weber et al., 2019).

### 2.3 Experimental measures

#### 2.3.1 Blood draw

Blood was drawn by venipuncture in the morning after at least 8 h of fasting. Blood was processed immediately afterward by laboratory staff outside the isolation module. The samples were centrifuged and plasma isolated, aliquoted, and stored at -80°C until transported to the laboratory of the PI. Immunoassay procedures were performed to evaluate the BDNF, IGF-1, cortisol, and melatonin levels in participants’ plasma. BDNF levels were evaluated using the Human Neurodegenerative Bead Panel 3 (EMD Millipore’s MILLIPLEX MAP) and biotinylated antibody detection. Cortisol levels were evaluated with micro-particle immunoassays *via* the Architect Cortisol kit. Levels of IGF-1 were evaluated *via* a solid-phase enzyme labeled chemiluminescent immuno-metric assay (IMMULITE 2000 IGF-1 kit). Melatonin concentration was analyzed from blood serum by I–Radioimmunoassay (Labor Diagnostika Nord GmbH & Co. KG, Nordhorn, Germany).

#### 2.3.2 Cognitive performance

Cognitive performance was assessed using two different cognitive test batteries. The “Cognition” test battery by Basner et al. (2015); Moore et al. (2017) is part of the Behavioral Health and Performance Standard Measures of NASA core data. The “brain games” test battery also aimed to investigate the cognitive performance and has been used in previous isolation studies (Schneider et al., 2013; Abeln et al., 2015; Weber et al., 2019). For both test batteries, participants were instructed to perform the tasks as fast and as accurate as possible. All tasks were performed for familiarization during the training session prior to isolation. Participants were asked to repeat the tests three times to familiarize with each test.

#### 2.3.3 Cognitive test battery

A detailed description of the cognitive test battery consisting of 10 cognition tests can be found in Basner et al., 2015; Moore et al., 2017. Here, we provide a brief overview of each test, where the tests were always performed in the order listed below. Cognition was administered on a fourth generation iPad (Apple, California, United States). The Motor Praxis Task (MP) measured sensorimotor speed. The Visual Object Learning Task (VOLT) assessed the participants’ memory for complex figures. The Fractal 2-Back (F2B) test tested the working memory system. The Abstract Matching (AM) test is a validated measure of the abstraction and flexibility components of executive function. The Line Orientation Test (LOT) is a measure of spatial orientation. The Emotion Recognition Task (ERT) is a measure of facial emotion recognition. The Matrix Reasoning Test (MRT) is a measure of abstract reasoning and consists of increasingly difficult pattern-matching tasks. The Digit-Symbol Substitution Task (DSST) is a measure of complex scanning, visual tracking, and working memory. The Balloon Analog Risk Test (BART) is an assessment of risk-taking behavior. The Psychomotor Vigilance Test (PVT) is a measure of vigilant attention and has been well-established as a tool to detect acute and chronic sleep deprivation and circadian misalignment (Basner et al., 2011).

#### 2.3.4 Brain games

Cognitive performance was also assessed using three different cognitive tasks from a commercial brain game (lumosity.com) where participants were asked to perform on an iPad (Apple, California, United States). The memory matrix task tested the visuospatial working memory as well as spatial imagination. The speed match task assessed visuoperceptual attention as well as working memory and decision-making. The chalkboard challenge tested the executive function *via* mathematical problem-solving and quantitative reasoning. These same tests have been used in the previous confinement investigations (Schneider et al., 2013; Abeln et al., 2015) and described in detail (Weber et al., 2019).

#### 2.3.5 Actigraphy

To assess sleep–wake timing during isolation, actigraphy was continuously recorded at the non-dominant wrist throughout the interventions. All participants continuously wore a small, light-weight activity-recording device (Actiwatch-L (AWL); MiniMitter/Phillips Respironics, Bend, OR). Actigraphy data were collected in 1-min epochs and scored in 2-min epochs. Sleep and wake periods were analyzed for each day and averaged for a 7-day period prior to the data collection days. Total sleep time, onset latency, sleep efficiency, wake after sleep onset (WASO), number of awakenings, and fragmentation index were compared between the groups. The fragmentation index was calculated as the product of % mobility and % 1 min immobility. % mobility is the relative number of minutes with one or more movements divided by time in bed. % 1 min immobility is defined as the phases of 1 min immobility relative to the total number of immobility phases of all durations. These data were exported from Philips Respironics Actiware software.

#### 2.3.6 Sleep questionnaire

The subjective Sleep and Awakening Quality (SSA) questionnaire (Saletu et al., 1987; Rosipal et al., 2013; Paiva et al., 2021) is composed of three subsets of questions: SSA1 includes questions regarding sleep quality, SSA2 regarding sleep awakening quality, and SSA3 regarding somatic complaints. Each question had to be answered based on the past week on a four-point Likert scale (“no”, “slightly”, “moderately”, and “very much”), with a score ranging from 1 to 4. A total score of 28 for SSA1, 32 for SSA2, and 20 for SSA3 could be achieved, where SSA4 represented the total sum score of all the subsets.

#### 2.3.7 State of affect–PANAS-X

The PANAS-X is a widely used self-report measure (Watson and Clark, 1999), which is valid and reliable (Watson, 1988a; Watson, 1988b; Watson and Walker, 1996) to assess the specific distinguishable states that emerge from the general dimension of positive and negative emotional experiences. The general positive affect (GPA) and the general negative affect (GNA) have been identified in both intra- and inter-individual analyses and emerge in a consistent way across sets, time frames, response formats, languages, and cultures. The PANAS-X is a 60-item expended version of the PANAS. In addition to the two original higher-order scales, the PANAS-X measures 11 specific affects: fear, sadness, guilt, hostility, shyness, fatigue, surprise, self-assurance, joviality, attentiveness, and serenity. Participants had to rate adjectives based on their own feelings on a five-point Likert scale from 1 (“not at all”) to 5 (“extremely”). The results of all subscales were computed *via* the sum of the respective items. Participants were asked to complete an electronic version of the PANAS-X by indicating their average feeling during the past week.

#### 2.3.8 Electro-encephalography (EEG)

EEG activity was recorded using an electrode cap (ActiCap EEG Active Electrode System combined with V-Amp Amplifier, Brain Products GmbH, Gilching, Germany) with Ag/AgCl electrodes located at 16 scalp sites (Fp1, Fp2, Fz, F3, F4, F7, F8, C3, Cz, C4, P3, Pz, P4, O1, Oz, and O2) based on an international 10–20 system (Jasper 1958). The conductivity of the electrodes was enhanced by adding gel (Super-Visc, EasyCap GmbH, Herrsching, Germany). The sample frequency was set at 500 Hz. Crew members of the isolated groups have been trained to mount the EEG cap and assist recording pairwise in a training session prior to each mission. Brain cortical activity was continuously measured for 5 min in a relaxed, upright seated position with the eyes closed. Participants were asked to concentrate on themselves, relax, and refrain from movement. The surrounding was kept as quiet as possible.

EEG data were analyzed with Brain Vision Analyzer 2 (Brain Vision Analyzer software, Brain Products, Munich, Germany). Raw EEG data were filtered utilizing a Butterworth zero-phase filter including a notch filter at 50 Hz for the CTRLs and at 60 Hz for the ISO groups. Low cut-off and high cut-off were set at 1 Hz and 30 Hz, respectively. The 5-min recordings were segmented into segments of 2,000 ms. A semi-automatic artifact rejection algorithm was applied on each segment. The segments were marked and removed if the difference between the minimum and maximum amplitude in a single segment exceeded 100 μV. The maximal allowed voltage step was set to 50 μV/ms. The lowest allowed activity was set to 0.5 μV. If an artifact was detected, the algorithm marked the event 200 ms before and 200 ms after the exact artifact occurred to control for the source of noise. Marked segments were untagged manually only if large portions of the clearly visible alpha were marked automatically due to their passing of the applied threshold. In addition, independent component analysis (ICA) was used to remove ocular, pulse-related, and movement-related artifacts not being coded by the semi-automatic artifact algorithm. Data were then baseline-corrected over 2,000 ms. Insufficient signal quality parameters (artifacts, slow voltage drifts, and impedance exceeding 10 kΩ) were excluded from data comparison. The remaining data were fast Fourier-transformed into delta (1–4 Hz), theta (4–8 Hz), alpha (8–12 Hz), and beta (12–30 Hz) frequency bands and exported as µV^2^ averaged over all remaining segments. The alpha peak (maximum value between 8 and 12 Hz) was calculated for each channel and an average of all channels for global alpha peak assessment (Weber et al., 2020).

#### 2.3.9 Exercise data

Physical exercise during intervention periods of ISO_100_, ISO_50_, and CTRL_Ex_ was monitored with training logs and heart rate (HR) monitors (Polar M400, Polar Electro GmbH, Büttelborn, Germany). Self-reported training logs provided data on the amount of sessions performed, total session duration, and the rate of perceived exertion (RPE) during each session. During each exercise session, second by second, HR data were collected and averaged over the duration of which the exercise was performed. These averaged HR and RPE data of each training session were averaged for all training sessions within 7 days prior to data collection and used for data comparison. HR and RPE were combined for strength exercise and endurance exercise sessions.

#### 2.3.10 Physical exercise testing of CTRL_NonEx_

To assess the cardiorespiratory fitness in the CTRL_NonEx_ group, an incremental bicycle exercise test until voluntary exertion before and after the intervention was performed. The work rate (WR) protocol on a semi-recumbent (backrest at 45°, leg exercise device at 42° from the horizontal) cycle ergometer (Lode Angio, Groningen, Netherlands) consisted of 5 min of rest, 5 min of 30 W, a 10-min phase of pseudo-randomly changing WRs of 30 W and 80 W, a 5-min period of 80 W, and 1 min of 100 W, followed by an incrementally increasing WR of 25 W each minute until voluntary exertion. Gas exchange was measured breath-by-breath, using a metabolic cart (Metalyzer 3B, Cortex Biophysik GmbH, Leipzig, Germany) and heart rate was assessed *via* ECG (CustoGuard belt 3, CustoMed, Ottobrunn, Germany). Prior to each test, the metabolic cart was calibrated according to the manufacturers’ guidelines. Peak oxygen uptake ( $\dot{V}$ O2 peak) was determined as the average of the last 30 s prior to test termination and used as a marker of cardiorespiratory fitness.

### 2.4 Statistical analysis

All data were normally distributed, assessed with the Kolmogorov–Smirnov Test. One-way (group) ANOVA was used to compare the age, height, weight, and BMI of all participants. Two-factor (group*time) ANOVA for repeated measures was used to compare the blood, EEG, actigraphy, cognitive test battery data, training logs, HR, and RPE of the four groups (ISO_50_, ISO_100_, CTRL_Ex_, and CTRL_NonEx_) for the two time points during each intervention (T1 and T2). Significant main effects and interactions were followed up with Bonferroni *post hoc* comparisons adjusted for multiple testing comparisons. Effect sizes (partial eta squared: η2 partial) were calculated and showed in all tables. Non-parametric tests were used to compare SSA and PANAS-X, where Mann–Whitney-U-test was used for group comparison and Kruskal–Wallis test for time comparisons. To assess the change in cardiorespiratory fitness for CTRL_NonEx_, a *t*-test was used. Statistical significance was set at *p* < .05. Statistical analyses were performed with Statisitca 7.1 (StatSoft).

### 2.5 Results

The participants of the CTRL group are slightly but significantly younger than those in the ISO group, whereas participant characteristics are similar between groups (Table 1).

#### 2.5.1 Blood marker

**FIGURE 1:**
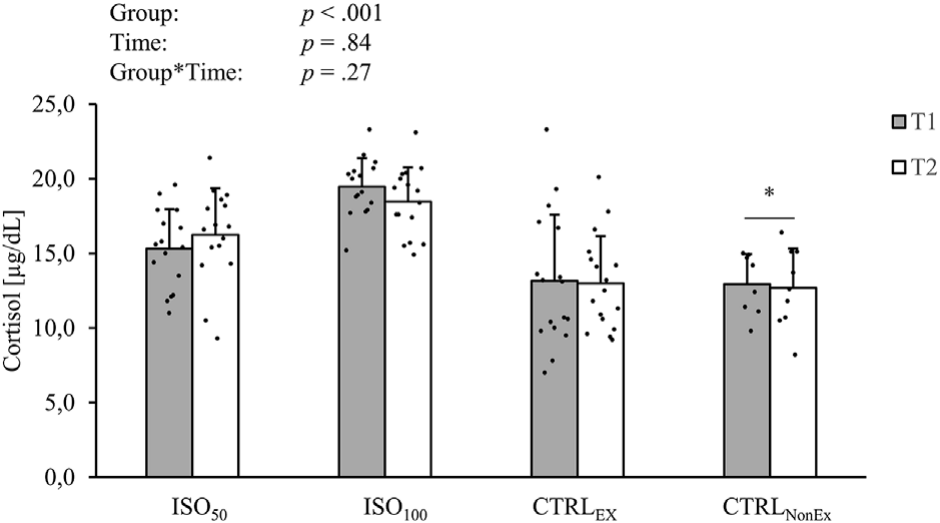
Absolute levels of intravenous cortisol during the different interventions. *p-*values represent ANOVA results. Data are displayed as mean ± standard deviation (SD). Filled circles display individual data points. * indicates a significant difference between the CTRL_NonEx_ group and the other groups (*p* < .05).

An overview of the morning (1 h after awaking) BDNF, IGF-1, cortisol, melatonin, adrenalin, and nor-adrenalin levels during ISO and CTRL periods is shown in Table 2. Blood markers before each intervention are shown in Supplementary Table S1. Cortisol levels are significantly higher in the isolated groups than in the non-isolated groups (Figure 1), but cortisol level was not different between groups prior to the interventions (Supplementary Table S1). BDNF was reduced across all groups over time (*p* < .05). Melatonin showed a reduction from T1 to T2 in the CTRL_NonEx_ group only (*p* < .05). IGF-1 increased over time (*p* < .01).

**TABLE 2:** Blood concentrations/factors during the interventions.

*Variable*	*T1*				*T2*				*G*			*T*			G*T		
	*ISO*_*100*_	*ISO*_*50*_	*CTRL*_*Ex*_	*CTRL*_*NonEx*_	*ISO*_*100*_	*ISO*_*50*_	*CTRL*_*Ex*_	*CTRL*_*NonEx*_	F (df)	n_p_^2^	p	F (df)	n_p_^2^	P	F (df)	n_p_^2^	p
BDNF (ng/mL)	28 (24-31)	32 (27-37)	25 (23-28)	23† (17-30)	24 (21-28)	30 (26-34)	25 (22-28)	22* (16-28)	3.03 (3,55)	.14	.03	4.57 (1,55)	.07	.05	.91 (3,55)	.05	.44
IGF-1 (µg/L)	184 (158-209)	166 (141-190)	147 (117-176)	160 (146-174)	199 (173-226)	178 (150-205)	158 (132-184)	175 (151-200)	1.81 (3,52)	.09	.16	7.39 (1,52)	.12	.01	.27 (3,52)	.02	.84
Melatonin (µg/dL)	22 (16-29)	37 (25-50)	18 (11-26)	50 ^e^ (13-87)	23 (14-32)	39 (28-51)	18 (13-23)	40^e^ ^,pre^ (18-62)	3.65 (3,53)	.17	.01	1.17 (1,53)	.02	.12	1.91 (3,53)	.10	.03
Adrenalin (µg/L)	49 (36-61)	68 (52-35)	83 (54-111)	43 (26-60)	47 (38-55)	43 (35-50)	90 (22-158)	48 (29-66)	1.97 (3,53)	.10	.12	.17 (1,53)	.01	.50	.67 (3,53)	.04	.60
Noradrenalin (µg/L)	506 (406-607)	477 (397-557)	376 (276-476)	282^†^ (187-376)	431 (360-503)	382 (298-466)	322 (268-376)	299^†^ (167-432)	3.66 (3,54)	.17	.01	6.29 (1,54)	.10	.01	.69 (3,54)	.04	.56

Data represent the responses for the time points at the beginning of the intervention (T1) and the end of the intervention (T2) and are displayed as mean and 95% confidence interval. F: F-value; df: degree of freedom; *ƞ*_*p*_^*2*^: Effect size (partial eta squared); G: group; T: time; BDNF: brain-derived neurotrophic factor; IGF-1: insulin-like growth factor-1; ISO_100_: isolated group 100% exercise; ISO_50_: isolated group 50% exercise; CTRL_Ex_: non-isolated exercise control group; CTRL_NonEx_: non-isolated non-exercise control group; * difference to ISO_50_ (*p* < .05).

^a^ Difference to CTRL_Ex_ (*p* < .05).

^pre^ Difference to T1 (*p* < .05); † Difference to ISO_100_ (*p* < .05).

#### 2.5.2 Sleep

##### 2.5.2.1 Actigraphy

**FIGURE 2:**
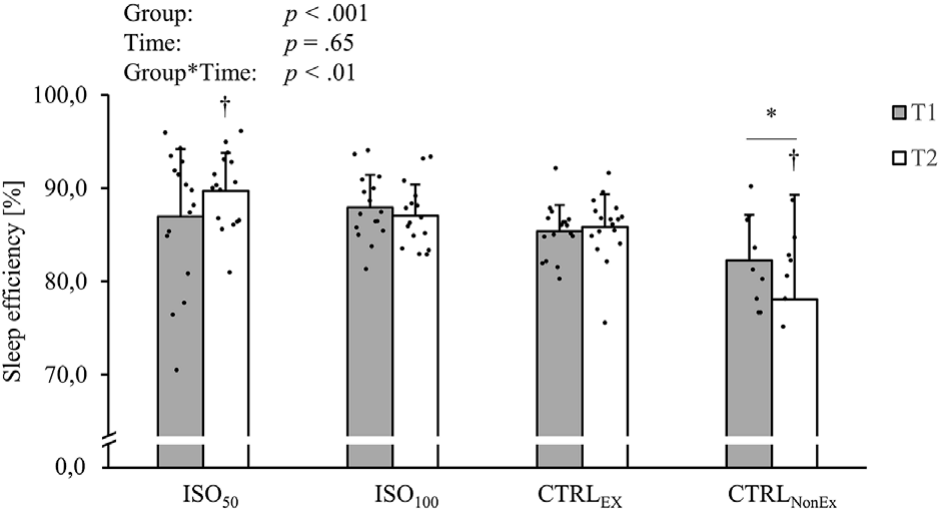
Responses of sleep efficiency during the different interventions. *p*-values represent ANOVA results. Data are displayed as mean ± SD. Filled circles display individual data points. * indicates a significant difference between CTRL_NonEx_ and the other groups (*p* < .05); † indicates the significant difference to T1 (*p* < .05).

Sleep results, recorded with actigraphy, are presented in Table 3. Total sleep time (minutes) showed a significant group effect (*p* < .001), where the ISO_50_ group had less sleep time than ISO_100_ (*p* < .001) and CTRL_Ex_ (*p* < .001) but not the CTRL_NonEx_ group (*p* = .031). The preserved sleep quality was even shown in ISO_50_, where the time of total sleep at night was restricted to 5 h. Sleep time was also reduced over the duration of the intervention (*p* < .001) and showed a group*time interaction (*p* < .001), where in the CTRL_NonEx_ and in ISO_50_ groups, the sleep time was lower at T2 than at T1 (*p* < .001). Sleep onset latency showed a group effect (*p* < .01), where ISO_50_ was faster than CTRL_Ex_ (*p* = .01) and CTRL_NonEx_ (*p* < .01), but not ISO_100_ (*p* = .05). There were no time effects (*p* = .96) or interactions for sleep onset latency (*p* = .34). Sleep efficiency is shown in Figure 2, where ISO_50_ showed an increase and CTRL_NonEx_ showed a reduction in sleep efficiency from T1 to T2. The number of awakenings showed a group effect (*p* < .001), where ISO_50_ showed lower numbers of awakenings than CTRL_Ex_ (*p* < .01) and CTRL_NonEx_ (*p* < .01), but not ISO_100_ (*p* = .09). The number of awakenings gradually reduced over the period of the intervention (*p* < .001), and the significant interaction (*p* < .001) indicates a reduction in ISO_50_ from T1 to T2 (*p* < .001) only. The fragmentation index was not different between groups (*p* < .42) and times (*p* = .07) but showed a significant group*time interaction (*p* < .01), where only ISO_50_ showed a reduction from T1 to T2 (*p* < .01).

**TABLE 3:** Sleep data (actigraphy).

*Variable*	*T1*				*T2*				*G*	*T*				G*T			
	*IS O*_*100*_	*IS O*_*50*_	*CTRL*_*Ex*_	*CTRL*_*NonEx*_	*IS O*_*100*_	*IS O*_*50*_	*CTRL*_*Ex*_	*CTRL*_*NonEx*_	*F (df)*	*n*_*p*_^*2*^	*p*	*F (df)*	*n*_*p*_^*2*^	*p*	*F (df)*	*n*_*p*_^*2*^	*p*
Total sleep time (minutes)	386 (373–399)	380^a†^ (360–400)	405 (388–422)	413 (363–464)	411 (396–427)	296^a^ (290–302)	403 (386–420)	381^†^ (328–434)	6.61 (3,54)	.27	<.01	23.14 (1,54)	.29	<.01	15.88 (3,54)	0.47	<.01
Onset sleep latency (minutes)	10 (8–13)	8^a^ (5–11)	14 (10–17)	13 (5–20)	14 (8–19)	5^a^ (3–7)	12 (10–15)	16 (10–22)	6.46 (3,53)	.27	<.01	<.01 (1,53)	<.01	.96	1.10 (3,53)	0.06	.34
WASO (minutes)	36 (30–42)	38 (29–47)	45 (39–50)	60^i^ (47–74)	40 (33–47)	23^†^ (18–28)	42 (37–48)	57^i^ (36–79)	7.68 (3,53)	.30	<.01	13.38 (1,53)	.20	<.01	11.07 (3,53)	0.39	<.01
Awaking (number)	21 (18–25)	20 (16–23)	26 (23–28)	30^f^ (24–35)	24 (20–27)	13^†^ (11–16)	24 (22–27)	25^f^ ^,†^ (19–31)	8.91 (3,53)	.33	<.01	17.07 (1,53)	.24	<.01	10.84 (3,53)	0.38	<.01
Fragmentation index	28 (24–31)	29 (24–35)	32 (30–35)	33 (26–39)	30 (26–33)	25^†^ (21–29)	31 (28–34)	31 (22–40)	1.23 3,53)	.06	.42	3.44 (1,53)	.06	.07	5.36 3,53)	0.23	<.01

Data are displayed as mean and 95% confidence interval for the time points at the beginning of the intervention (T1) and the end of the intervention (T2). F: F-value; df: degree of freedom; *ƞ*_*p*_^*2*^: effect size (partial eta squared); G: group; T: time; WASO: wake after sleep onset. ISO_100_: isolated group 100% exercise; ISO_50_: isolated group 50% exercise; CTRL_Ex_: non-isolated exercise control group; CTRL_NonEx_: non-isolated non-exercise control group.

^a^ indicates ISO_50_ different to other groups (*p* < .05).

^b^ CTRL_Ex_, different to ISO_100_ and ISO_50_ (*p* <. 05).

^c^ ISO.^100^.

different to CTRL_Ex_ and CTRL_NonEx_ (*p* < .05).

^d^ CTRL_Ex_, different to ISO_100_ (*p* < .05).

^e^ ISO_100_ different to other groups (*p* <. 05).

^f^ CTRL_NonEx_ different to ISO_50_ (*p* <. 05).

^g^ CTRL_NonEx_ different to CTRL_Ex_ and ISO_50_ (*p* < .05).

^h^ CTRL_NonEx_ different to ISO_100_ (*p* < .05).

^i^ CTRL_NonEx_ different to other groups (*p* < .05); † different to T1 (*p* < .05).

#### 2.5.2 Sleep questionnaire

The subjective Sleep and Awakening Quality (SSA) questionnaire assessed sleep quality (SSA1), which showed a group difference (*p* < .001) with higher scores in the ISO_100_ (*p* < .001) and CTRL_Ex_ groups(*p* < .001) than in the ISO_50_ and CTRL_NonEx_ groups. ISO_50_ and CTRL_NonEx_ were not different from each other (*p* = .74). SSA1 did not show a time effect (*p* = .79) or a group*time interaction (*p* = .88). Awakening quality (SSA2) showed a group difference (*p* < .001) with higher scores in ISO_100_ (*p* < .001) and CTRL_Ex_ (*p* < .001) than in ISO_50_ and CTRL_NonEx_. ISO_50_ and CTRL_NonEx_ were not different between each other (*p* = .99). SSA2 did not show a time effect (*p* = .16) or a group*time interaction (*p* = .46). Somatic complaints (SSA3) were neither significant for the factor group (*p* = .06) and time (*p* = .36) nor the interaction (*p* = .72). The total scores (SSA4) showed a group effect (*p* < .001), where ISO_100_ was higher than ISO_50_ and CTRL_Ex_ (*p* < .001), but not to CTRL_NonEx_ (*p* = .34). There was no time effect for SSA4 (*p* = .72) or a group*time interaction (*p* = .98).

#### 2.5.3 Cognitive performance tests

##### 2.5.3.1 Cognition test battery

Overall reaction time (calculated as an average of all subtasks) showed an overall group effect (*p* < .01), where the ISO_100_ group showed a faster reaction time than the CTRL_Ex_ group. Overall, there was a time effect (*p* < .001), where the reaction time decreased from T1 to T2 and a group*time interaction (*p* = .04). Bonferroni *post hoc* analysis revealed that there was an improvement of reaction time in the ISO_50_ group from T1 to T2, where the reaction time decreased significantly (*p* = .01). Overall, the accuracy (calculated as an average of all subtasks) showed no group effect (*p* = .43) and no time effect (*p* = .17), but a group*time interaction (*p* = .01), where Bonferroni *post hoc* analysis did not show any differences. Results for each subtest of the cognition test battery are presented in Table 4 for reaction time; Table 5 for accuracy. Psychomotor vigilant attention (PVT) showed a significant group*time interaction for reaction time (Table 4) and *posthoc* analysis showed a reduction of reaction time only in the CTRL_NonEX_ group over the duration of the intervention, where PVT reaction time was lower at T2 than at T1 (*p* < .01).

**TABLE 4:** Cognitive test battery during each intervention—reaction time.

*Test*	*T1*				*T2*			*G*				*T*			G*T		
*Reaction time*	*IS O*_*100*_	*IS O*_*50*_	*CTRL*_*Ex*_	*CTRL*_*NonEx*_	*IS O*_*100*_	*IS O*_*50*_	*CTRL*_*Ex*_	*CTRL*_*NonEx*_	*F (df)*	*n*_*p*_^*2*^	*p*	*F (df)*	*n*_*p*_^*2*^	*p*	*F (df)*	*n*_*p*_^*2*^	*p*
MPT (s)	.46 (.43–.49)	.80 (.72-.89)^a^	.50 (.47–.52)	.48 (.45–.52)	.47 (.46–.49)	.75 (.70-.80)^a^	.47 (.45–.49)	.46 (.41–.51)	57.9 (3,55))	.76	<.01	4.19 (1,55)	.07	.04	2.24 (3,55)	.11	.09
VOLT (s)	1.50 (1.37–1.63)	1.76 (1.54–1.97)	2.15 (1.82–2.47)^b^	1.78 (1.44–.213)	1.36 (1.22–1.51)	1.28 (1.19–1.37)	1.97 (1.50–2.43)^b^	1.34 (.89–1.79)	4.91 (3,55)	.21	<.01	28.08 (1,55)	.34	<.01	2.40 (3,55)	.12	.07
F2B (s)	.66 (.60–.71)	.69 (.62–.76)	.61 (.57–.65)	.59 (.55–.63)	.60 (.56–.65)	.60 (.55–.66)	.57 (.49–.65)	.63 (.58–.68)	.99 (3,54)	.05	.38	5.84 (1,54)	.10	.02	2.43 (3,54)	.07	.08
AMT (s)	1.83 (1.51–2.15)	2.14 (1.86–2.42)	2.23 (1.94–2.53)	1.59 (1.13–2.04)	1.58 (1.16–2.00)	1.92 (1.54–2.30)	2.09 (1.80–2.38)	1.50 (.57–2.44)	2.42 (3,55)	.12	.07	3.72 (1,55)	.06	.06	.15 (3,55)	.01	.92
LOT (s)	4.87 (3.44–6.30)	5.66 (5.08–6.24)	7.08 (5.32–8.85)	6.23 (4.93–7.54)	4.82 (3.42–6.21)	5.34 (4.46–6.22)	6.30 (5.22–7.38)	5.79 (3.63–7.95)	2.13 (3,55)	.10	.10	1.12 (1,55)	.02	.29	.19 (3,55)	.01	.90
ERT (s)	1.49 (1.36–1.63)^c^	1.94 (1.76–2.12)	2.35 (2.03)	2.29 (1.78–2.80)	1.50 (1.30–1.70)^c^	1.67 (1.51–1.84)	2.05 (1.75–2.36)	2.14 (1.54–2.74)	7.46 (3,55)	.29	<.01	8.71 (1,55)	.14	<.01	1.62 (3,55)	.08	.19
MRT ([s)	6.19 (5.13–7.24)	9.51 (8.32–10.69)^d^	9.26 (7.81–10.72)	8.50 (6.32–10.68)	6.99 (5.53–8.46)	6.49 (5.36–7.63)^d^ ^,†^	9.58 (7.80–11.37)	6.61 (4.60–8.61)	3.46 (3,55)	.16	.02	6.93 (1,55)	.29	.01	7.51 (3,55)	.30	<.01
DSST (s)	.89 (.84-.94)^e^	1.30 (1.16–1.44)	.92 (.89–.96)	.98 (.89–1.06)	.88 (.83-.94)^e^	1.18 (1.07–1.29)^†^	.91 (.88–.95)	.96 (.88–1.04)	17.27 (3,55)	.48	<.01	19.68 (1,55)	.26	<.01	11.10 (3,55)	.38	<.01
BART (s)	.24 (.21–.27)	.35 (.25–.44)	.37 (.29–.46)	.36 (.28–.43)	.24 (.19–.28)	.31 (.22–.41)	.34 (.27–.40)	.31 (.26–.37)	2.23 (3,55)	.10	.09	4.99 (1,55)	.08	.02	.05 (3,55)	.03	.68
PVT (s)	.24 (.23–.26)	.23 (.22–.25)	.24 (.23–.25)	.25 (.23–.27)	.23 (.21–.24)	.24 (.21–.27)	.24 (.22–.26)	.28 (.25-.31)^†^	1.84 (3,55)	.09	.15	3.55 (3,55)	.06	.06	5.52 (3,55)	.23	<.01

Data are displayed as mean and 95% confidence interval for the time points at the beginning of the intervention (T1) and the end of the intervention (T2). F: F-value; df: degree of freedom; *ƞ*_*p*_^*2*^: Effect size (partial eta squared); G: group; T: time; s: Seconds; MP: motor praxis; VOL: visual object learning; F2B: Fractal-2-Back; AM: abstract matching; LO: line orientation; DSS: digit symbol substitution; BAR: balloon analog risk; PV, psychomotor vigilance; ISO_100_, isolated group 100% exercise; ISO_50_, isolated group 50% exercise; CTRL_Ex_,, non-isolated exercise control group; CTRL_NonEx_, non-isolated non-exercise control group.

^a^ ISO_50_ different to other groups (*p* <. 05).

^b^ CTRL_Ex_, different to ISO_100_ and ISO_50_ (*p* <. 05).

^c^ ISO.^100^.

different to CTRL_Ex_ and CTRL_NonEx_ (*p* < .05).

^d^ CTRL_Ex_, different to ISO_100_ (*p* < .05).

^e^ ISO_100_ different to other groups (*p* < .05); † different to T1 (*p* < .05).

**TABLE 5:** Cognitive test battery during each intervention—accuracy.

*Test*	*T1*				*T2*				*G*			*T*				G*T	
*Accuracy*	*IS O*_*100*_	*IS O*_*50*_	*CTRL*_*Ex*_	*CTRL*_*NonEx*_	*IS O*_*100*_	*IS O*_*50*_	*CTRL*_*Ex*_	*CTRL*_*NonEx*_	*F (df)*	*n*_*p*_^*2*^	*p*	*F (df)*	*n*_*p*_^*2*^	*p*	*F (df)*	*n*_*p*_^*2*^	*p*
VOLT (%)	84 (79–89)	84 (80–88)	80 (73–87)	85 (79–91)	86 (81–90)	91 (86–96)	86 (82–90)	82 (72–92)	.93 (3,55)	.05	.43	3.13 (1,55)	.05	.08	1.58 (3,55)	.07	.20
F2B (%)	90 (87–93)	92 (90–94)	90 (88–92)	88 (84–91)^f^	92 (88–95)	94 (90–97)	89 (85–92)	84 (77–90)^f,†^	3.25 (3,55)	.15	.02	.39 (1,55)	<.01	.53	2.78 (3,55)	.13	.04
AMT (%)	69 (63–75)	73 (68–79)	70 (64–75)	57 (49–64)^g^	70 (63–77)	81 (75–87)	70 (63–77)	58 (54–62)^g^	6.64 (3,55)	.26	<.01	2.58 (1,55)	.04	.11	1.41 (3,55)	.07	.24
LOT (%)	40 (32–47)	41 (32–50)	49 (40–57)	55 (26–84)	48 (41–56)	40 (31–48)	46 (40–52)	48 (29–67)	.86 (3,55)	.04	.46	.03 (1,55)	<.01	.84	1.96 (3,55)	.10	.13
ERT (%)	63 (58–67)	60 (56–63)	62 (59–64)	61 (52–70)	63 (60–67)	69 (66–72)^†^	67 (61–72)	59 (52–66)	.83 (3,55)	.04	.48	7.19 (1,55)	.12	<.01	4.14 (3,55)	.19	.01
MRT (%)	74 (68–80)	70 (62–78)	68 (61–74)	59 (49–.69)	75 (69–81)	77 (67–87)	65 (58–72)	61 (48–75)	3.04 (3,55)	.15	.03	.70 (1,55)	.01	.40	.91 (3,55)	.05	.44
DSST (%)	99 (98–100)	97 (96–99)^a^	100 (100–100)	99 (99–100)	99 (99–100)	96 (94–97)^a^	99 (99–100)	99 (99–100)	15.00 (3,55)	.45	<.01	1.70 (1,55)	.03	.20	2.00 (3,55)	.10	.12
BART (score)	867 (803–932)	880 (805–956)	853 (774–933)	893 (837–950)	894 (828–961)	901 (815–988)	758 (628–890)	846 (763–930)	1.13 (3,55)	.06	.34	.99 (1,55)	.02	.32	1.79 (3,55)	.09	.15
PVT (%)	92 (88–95)	94 (91–97)	91 (88–94)	87 (83–91)^h^	94 (91–97)	89 (82–96)	90 (85–94)	81 (72–90)^h^	2.85 (3,55)	.13	.04	3.50 (1,55)	.06	.06	1.88 (3,55)	.09	.14

Data are displayed as mean and 95% confidence interval for the time points at the beginning of the intervention (T1) and the end of the intervention (T2). F: F-value; df: degree of freedom; *ƞ*_*p*_^*2*^: effect size (partial eta squared); G: group; T: time; visual object learning; F2B: Fractal-2-Back; AM: abstract matching; LO: line orientation; DSS: digit symbol substitution; BAR: balloon analog risk; PV, psychomotor vigilance; ISO_100_: isolated group 100% exercise; ISO_50_: isolated group 50% exercise; CTRL_Ex_: non-isolated exercise control group; CTRL_NonEx_ non-isolated non-exercise control group.

^a^ ISO_50_ different to other groups (*p* < .05).

^f^ CTRL_NonEx_ different to ISO_50_ (*p* < .05).

^g^ CTRL_NonEx_ different to CTRL_Ex_ and ISO_50_ (*p* < .05); h CTRL_NonEx_ different to ISO_100_ (*p* < .05); † Different to T1 (*p* < .05).

##### Brain games

Mental arithmetic (chalkboard challenge) did not show a difference in the scores between groups (*p* = .63), times of measurement (*p* = .15), or a significant group*time interaction (*p* = .63) during the intervention periods. Working memory and decision-making (speed match) were not different between groups (*p* = .32), but the score increased over the duration of the interventions (*p* < .01), which was consistent for all groups (*p* = .49). There was a group difference for working memory (memory matrix) (*p* = .02), where CTRL_NonEx_ was lower than ISO_50_ (*p* = .04), but not different to the other groups. Working memory scores increased over the duration of the intervention (*p* < .001); however, there was no significant group*time interaction (*p* = .10).

#### 2.5.4 State of affect

##### General negative affect (GNA) and general positive affect (GPA)

**FIGURE 3:**
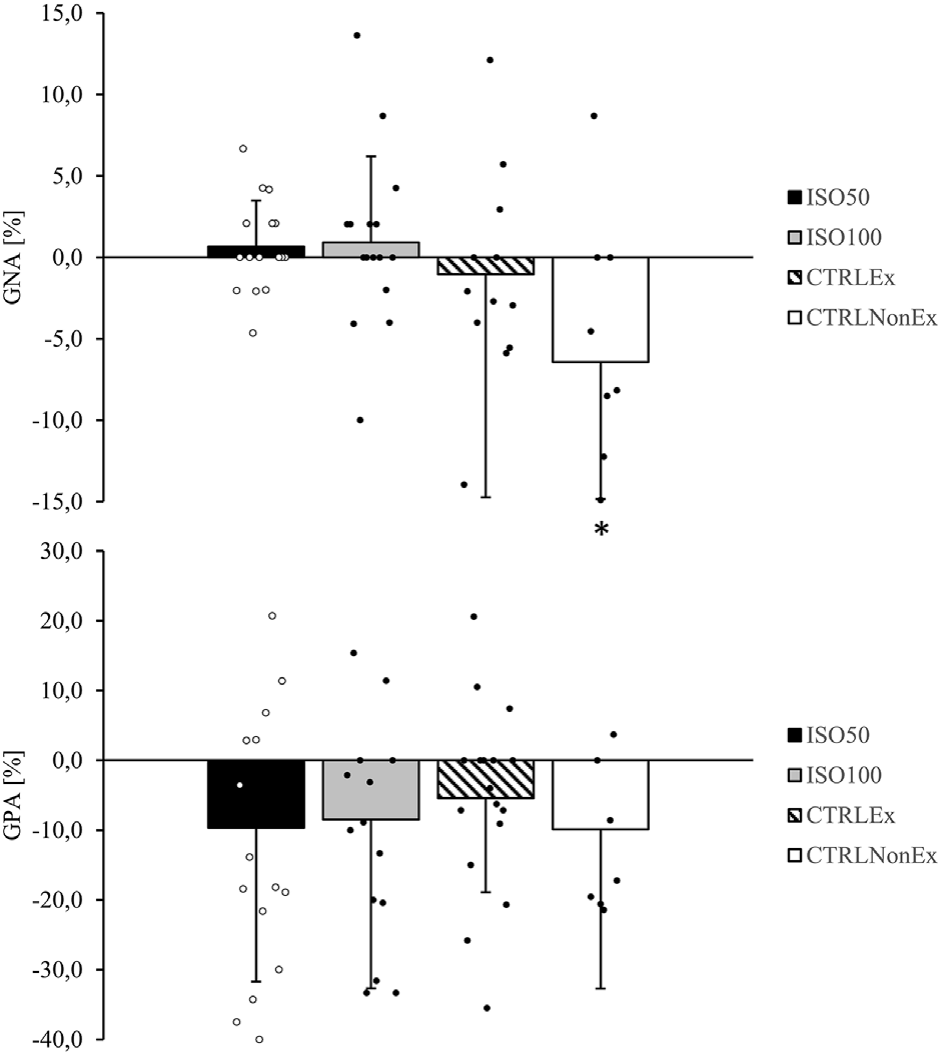
Responses of general negative affect (GNA) and general positive affect (GPA) from T1 relative to T2 during the different interventions. *p-*values represent ANOVA results. Data are the relative change from T1 to T2 as mean ± SD. Open and filled circles display individual data points. Symbols are used to show significant effects between groups. * indicates a significant difference between the CTRL_NonEx_ group and the other groups (*p* < .05).

General negative affect showed a group difference (*p* < .001), where CTRL_Ex_ was lower (*p* < .01) than ISO_100_, ISO_50_, and CTRL_NonEx_, which is an indicator for distress, calmness, and serenity (Watson, 1988a). GNA remained unchanged over time (*p* = .06), and no group*time interaction (*p* = .08) was observed. GPA showed a group effect (*p* < .01) and was lower in CTRL_NonEx_ than in CTRL_Ex_ (*p* = .10), ISO_100_ (*p* = .04), and ISO_50_ (*p* = .07). GPA decreased over time (*p* < .001), but there was no significant group*time interaction (*p* = .55). Analysis of GPA subgroups showed a significant reduction from T1 to T2 in joviality (*p* = .01) and attentiveness (*p* < .01), whereas subgroups’ self-assurance (*p* = .23), serenity (*p* = .35), and surprise (*p* = .94) remained unchanged. The relative change from T1 to T2 for GNA and GPA is shown in Figure 3.

#### 2.5.5 Electroencephalography (EEG)

Results for frequency band analysis of alpha, beta, gamma, and theta frequency bands are shown in Table 6 and revealed no differences between the groups and no changes over time. Theta frequency showed a significant group*time interaction, but Bonferroni correction could not detect significant *post hoc* differences. Alpha peak frequency also showed no difference between the groups and remained unchanged over time (Table 6).

**TABLE 6:** Resting frequency band analysis during each intervention.

*Variable*	*T1*				*T2*				*G*			*T*			G*T		
	*IS O*_*100*_	*IS O*_*50*_	*CTRL*_*Ex*_	*CTRL*_*NonEx*_	*IS O*_*100*_	*IS O*_*50*_	*CTRL*_*Ex*_	*CTRL*_*NonEx*_	*F (df)*	*n*_*p*_^*2*^	*p*	*F (df)*	*n*_*p*_^*2*^	*p*	*F (df)*	*n*_*p*_^*2*^	*p*
alpha (µV^2^)	1.14 (.83–1.46)	1.15 (.80–1.50)	1.03 (.85–1.22)	1.45 (1.13–1.78)	.99 (.60–1.38)	1.23 (.86–1.59)	.91 (.65–1.17)	1.37 (1.00–1.74)	1.24 (3,55)	.06	.30	1.73 (1,55)	.03	.19	.99 (3,55)	.05	.40
beta (µV^2^)	.22 (.17–.26)	.24 (.20–.27)	.26 (.24–.29)	.24 (.21–.28)	.22 (.17–.27)	.23 (.19–.27)	.26 (.24–.29)	.24 (.21–.28)	1.22 (3,55)	.06	.31	.33 (1,55)	<.01	.56	.13 (3,55)	.01	.94
delta (µV^2^)	1.31 (.90–1.71)	1.40 (.89–1.91)	1.63 (1.27–2.00)	.98 (.52–1.44)	1.37 (.87–1.88)	1.34 (.82–1.86)	1.81 (1.40–2.21)	.87 (.35–1.39)	1.86 (3,55)	.09	.14	.05 (1,55)	<.01	.81	.63 (3,55)	.03	.59
theta (µV^2^)	.10 (−.12–.31)	.26 (.09–.43)	.16 (.07–.25)	.00 (−.16–.17)	.26 (−.02–.53)	.17 (.02–.33)	.20 (.08–.33)	.02 (−.28–.32)	.75 (3,55)	.04	.41	1.32 (1,55)	.02	.56	3.53 (3,55)	.16	**.02**
alpha-peak (µV^2^)	3.15 (.92–5.37)	2.85 (1.09–4.60)	4.58 (.98–8.18)	5.24 (1.46–9.02)	3.03 (1.00–5.05)	2.57 (.62–4.52)	3.45 (1.85–5.05)	4.64 (1.80–7.48)	.61 (3,55)	.04	.60	1.77 (1,55)	.03	.19	.34 (3,55)	.02	.79

Data are displayed as mean and 95% confidence interval for the time points at the beginning of the intervention (T1) and the end of the intervention (T2). F: F-value; df: degree of freedom; *ƞ*_*p*_^*2*^: effect size (partial eta squared); G: group; T: time; ISO_100_: isolated group 100% exercise; ISO_50_: isolated group 50% exercise; CTRL_Ex_: non-isolated exercise control group; CTRL_NonEx_: non-isolated non-exercise control group.

#### 2.5.6 Exercise data

##### 2.5.6.1 Peak oxygen uptake (VO_2peak_) CTRL_NonEx_

Peak oxygen uptake in the CTRL_NonEx_ group was lower (*p* < .05) after the intervention (34.9 ± 6.8, mL/kg/min) than before the intervention (38.3 ± 5.6, mL/kg/min). Exercise data of the ISO_50_ group were published in detail by Koschate et al. (2021), which showed that VO_2peak_ of the ISO_50_ group remained unchanged over the duration of the isolation.

##### 2.5.6.2 Heart rate and training logs

As expected, the total number of physical exercise sessions was higher for ISO_100_ (28 ± 3 sessions, *p* < .001) and CTRL_Ex_ (25 ± 7 sessions, *p* < .001) than for ISO_50_ (17 ± 5 sessions). The average duration of the sessions was similar between ISO_50_ (58 ± 12, min) and ISO_100_ (60 ± 0, min), but longer for CTRL_Ex_ (105 ± 31, min, *p* < .001). The average heart rate response during exercise was similar (*p* = .66) between ISO_50_ (123 ± 12, bpm), ISO_100_ (121 ± 12, bpm), and CTRL_Ex_ (119 ± 11, bpm) groups. This is in line with the average rate of perceived exhaustion (RPE) for all training sessions, which was similar (*p* = .23) between ISO_50_ (12 ± 1), ISO_100_ (13 ± 2), and CTRL_Ex_ (13 ± 1) groups.

## 3 Discussion

This study aimed to investigate the effect of physical exercise during short-term isolation including its effects on sleep, state of affect, cognitive function, neurotrophic factors, and brain cortical activation. Cortisol, traditionally seen as a marker of stress, was significantly increased during isolation with regular exercise training without impairments in sleep efficiency and sleep quality of the isolated groups, ISO_100_ and ISO_50_. Positive and negative states of affect were not negatively influenced by isolation up to 45 days. Cognitive function, brain cortical activation, and neurotrophic factors were also preserved. The CTRL_Ex_ also did not show any deterioration. However, the absence of exercise over the duration of 30 days in a non-isolated control group (CTRL_NonEx_) showed a reduction in cardiorespiratory fitness and impairments in sleep quality, affect state, and cognitive function.

### 3.1 Effect of isolation on cortisol

Consistent with previous reports (Choukèr et al., 2002; Jacubowski et al., 2015) and as hypothesized, we observed an increase in cortisol levels during isolation. This is an accepted biological stress response during isolation, characterized by the synchronized activity of the sympathetic nervous system (SNS) and the hypothalamic–pituitary–adrenal (HPA) axis. Activation of the SNS results in the release of catecholamine, adrenaline, and noradrenaline by the locus coeruleus and the secretion of salivary alpha-amylase (Nater et al., 2005), while slower activation of the HPA axis triggers the release of the glucocorticoid hormone cortisol from the adrenal cortex (Ulrich-Lai and Herman, 2009). Once activated, these systems play a role in the adaptive physiological, state of affect, cognitive, and behavioral responses to stress (de Kloet et al., 2005). Interestingly, although levels of cortisol were increased during isolation, we did not observe impairments in mood and cognitive function. During space flights, cortisol also increases in a pattern associated with mission duration (Stowe et al., 2011). As these high levels of cortisol during isolation did not negatively impair brain function, mood, and sleep, but impairments in brain function mood and sleep were observed with the absence of exercise, we propose that cortisol response might have preserved central nervous system function. It might act as a protective mechanism for the brain, which may have resulted in the maintenance of cognitive function, brain cortical activation, mood, and sleep.

### 3.2 Effect of isolation on sleep

Other than long-term isolation, where impairments in sleep quality and quantity were observed (Mairesse et al., 2019), we could show that sleep was well-preserved during isolation, as we did not observe any sleep impairments in the ISO_100_ and ISO_50_ groups. Impairments in sleep were only observed in the CTRL_NonEx_ group. In line with the results on sleep quality, melatonin secretion and psychomotor vigilance were also impaired in the CTRL_NonEx_ group. As hypothesized, we did not observe impairments in these factors in the isolated groups where exercise was performed regularly. We assume that exercise preserved sleep quality and compensated the negative effects of isolation. This is supported from the current literature of non-isolation investigations, showing that physical exercise preserves sleep quality (Kovacevic et al., 2018; Kline et al., 2021). Hence, sleep can be defined as an actively regulating process and can be seen as an acute reorganization of neuronal activity (Hobson, 2005). Our observation of brain cortical activation showed no impairments in the isolated groups, even in the ISO_50_ group, where sleep was restricted to only 5 h per night. For completeness, it has to be mentioned that brain cortical activation during daytime was also preserved in CTRL_NonEx_. Because Mairesse et al. (2019) showed impairments in sleep after long-term isolation, the positive sleep results we observed might be linked to the duration of isolation, where 30–45 days of isolation or absence of exercise might be too short to significantly impair sleep quality and associated psycho-physiological health.

### 3.3 Effect of isolation on the brain

We hypothesized that cognitive function would be reduced due to stress-related structural changes in the brain (Liston et al., 2009), and this cognitive decline was expected to be reflected by a decrease of neurotrophic factors BDNF and IGF-1, as these factors are highly associated with cognitive function (Saatman et al., 1997; Prokopova et al., 2017; Sungkarat et al., 2018). As discussed previously, cognitive performance was not affected by isolation, and our results of BDNF and IGF-1 did not show deteriorations during isolation. Although evidence suggested that a prolonged period of increased stress adversely affects neurotrophic factors (Licinio and Wong, 2002; Sävendahl, 2012), we could show that short-term isolation, here 30–45 days, does not affect the regulation and expression of neurotrophic factors. This is in line with our findings in cognitive function. The positive effect of exercise on neurotrophic factors has been consistently reported in the literature (Miranda et al., 2019). With regards to short-term isolation, it could be hypothesized that exercise contributed to the maintenance of BDNF levels during short-term isolation. The maintenance of neurotrophic factors during isolation might have also contributed to maintenance of cortical activation during isolation, where we observed no isolation-dependent impairments across the EEG alpha, beta, delta, and theta frequency band activity. Although the EEG showed high variability, this is consistent with previous results, where alpha and beta frequency bands were preserved until approximately 60 days of isolation and reduced thereafter (Schneider et al., 2010). Recently, we could show brain cortical deactivation during 4 months of isolation (Weber et al., 2020). In this study, the deactivation (e.g., decrease of alpha peak frequency) was not as comprehensive after 15 days as after 54 days and adjacent days, suggesting that prolonged isolation may lead to increasing sensory deprivation with an associated downregulation of brain activation. This indicates that the present isolation period of 30 or 45 days was possibly not long enough to obtain reduction in brain cortical activation. Further research is needed to clarify the timing of changes in brain cortical activation during isolation and whether the types of exercise (resistance, endurance, etc.) or the exercise mode (continuous, interval, etc.) play a role in this regard.

### 3.4 Effect of isolation on state of affect and the effect of exercise

Mood deteriorations are commonly reported during long-term isolation in space–analog environments (e.g., 9 months (Abeln et al., 2015)), where it is assumed that longer periods of isolation adversely affect the mood (Palinkas and Houseal, 2000; Schneider et al., 2010). The general positive affect scale showed a reduction across all groups, which might therefore represent an effect of repeated tests rather than impairments due to isolation or exercise. The general negative affect scale was well-preserved in the isolated groups as hypothesized but showed a reduction over time in the CTRL_NonEx_ group only, where VO_2peak_, an indicator of cardiorespiratory fitness, also showed a reduction. Koschate et al. (2021) recently showed that the VO_2peak_ of the ISO_50_ group remained unchanged over the duration of the isolation. Both the isolated and the non-isolated exercise groups were not negatively affected during the intervention, where exercise was performed regularly. However, when exercise was prohibited, as in the CTRL_NonEx_ group, the negative affect scale significantly reduced during the intervention. This is particularly important for isolation and confinement interventions, where the absence of exercise may cause even more severe mood impairments. Cognition and state of affect are strongly linked as the state of affect refers to the underlying experience of feeling and emotion. Positive emotions are a key psychological component and enhance the ability to cope with stressful events (Tugade and Fredrickson, 2004). This is important for cognitive performance, as the state of affect influences the interaction of an individual with stimuli including the cognitive scope and, thus, cognitive performance. The reduced psychomotor vigilance and working memory within the CTRL_NonEx_ group in parallel to the reduced negative affect support this assumption.

### 3.5 Effect of isolation on cognitive performance

When cortisol crosses the brain–blood barrier, the binding to glucocorticoid receptors in the brain occurs in particular abundance in the prefrontal cortex, hypothalamus, and hippocampus (Reul and de Kloet, 1985; Alderson and Novack, 2002) and alters neural functioning in those regions. Previous studies demonstrate that acute psychosocial stress (and consequent elevations in cortisol levels) negatively affects speed and accuracy in the performance of working memory tasks, especially at greater cognitive loads (Lupien et al., 1999; Wolf et al., 2001; Mizoguchi et al., 2004; Elzinga and Roelofs, 2005; Oei et al., 2006; Schoofs et al., 2008). The literature shows mixed results with a smaller group of studies, suggesting that such cortisol elevations improve, or may have no effect, on the working memory task performance (Kuhlmann et al., 2005; Oei et al., 2009; Weerda et al., 2010). This is in line with our observation, in which increased levels in cortisol in the isolated groups did not go along with the negative results in the working memory task. These results are particularly important for astronauts during space missions to ensure working memory function and therefore astronaut’s safety and space mission success. In addition to cortisol, sleep has been shown to influence cognitive performance. While Mairesse et al. (2019) showed impairments in sleep quality and in psychological vigilance for long-term isolation, we could show that sleep quality and psychomotor vigilance were not impaired in the isolated groups during short-term isolation. Interestingly, we observed impaired sleep and psychomotor vigilance in the CTRL_NonEx_ group, which supports our hypothesis that exercise might contribute to the maintenance of sleep quality and therefore cognitive performance. Psychomotor vigilance was maintained even when the total sleeping time was restricted, as for the ISO_50_ group. This preserved state of affect and cognitive performance in the isolated groups with regular physical activity is an important finding and of outmost importance for prescriptions and recommendations of isolation missions such as spaceflight or pandemic lockdown scenarios. Future research is needed to better understand the factors that negatively affect cognitive function during isolation and confinement and the contribution of physical exercise with a focus on the types of exercise, exercise modes, duration, and intensity.

### 3.6 Limitations

There are limitations in this study to consider. This study aimed to provide a holistic investigation of the effect of exercise during short-term isolation on psycho-physiological responses including links to cognitive performance, sleep, and underlying mechanisms. Generally, the number of participants in isolation studies is low and the number of implemented studies high for feasibility and economic reasons; therefore, it was our intention to combine two isolation campaigns to increase the number of participants in isolation to a total of 32 subjects. This led to different isolation periods, e.g., ISO_100_ 30 days and ISO_50_ 45 days, assessment time points, and differing imbedded tests and interventions during the isolation campaigns. NASA was responsible for recruitment of the isolated groups and the experimental design within HERA. The inclusion of a non-exercise control group in an isolated, confined, and controlled environment within HERA is an experimental design question. Before investigating a non-exercise group in an isolated environment, we first need to better understand the risks of restricting exercise during isolation. We, therefore, aimed to compare at least two different exercise protocols in isolation and in a non-isolated control group who stopped their exercise routines for the duration of the intervention to disentangle the effects of exercise. It exceeds the scope of this manuscript to consider all possible influencing factors, such as group cohesion or crew compatibility, and it remains difficult to determine the pure effect of isolation or the pivotal factor contributing to the high stress level. There is a need for further investigations, which systematically examine the effect of various factors of isolation and spaceflight to develop efficient countermeasures against neurobehavioral impairments.

### 3.7 Conclusion

In conclusion, psycho-physiological as well as brain functions were not impaired during short-term isolation of up to 45 days where physical exercise was performed regularly on a daily or every second day basis. The absence of exercise may contribute to manifesting deteriorations already within a short period of time (30 days), which were observed in the non-isolated non-exercising group. Exercise might function as a preserver of sleep quality and affect state, which possibly maintained neuro-psychological function and health, although high levels of stress occurred during isolation. We suggest that physical exercise is a key component during isolation to maintain brain health.

## Data Availability

The raw data supporting the conclusions of this article will be made available by the authors, without undue reservation.
