# Supplementary material for: Short-term isolation effects on the brain, cognitive performance, and sleep—The role of exercise
Source: Front Physiol. 2023 Jan 30;14:903072. doi: 10.3389/fphys.2023.903072 (PMC9927017; doi:10.3389/fphys.2023.903072)
Supplement: Supplementary file 1 [file Table1.DOCX]

# Supplementary Table 1 – Intravenous blood concentrations prior to and after each intervention

|  | **Pre intervention** | | | | **Group** |
| --- | --- | --- | --- | --- | --- |
|  | ***ISO_100_*** | ***ISO_50_*** | ***CTRL_Ex_*** | ***CTRL_NonEx_*** |  |
| Cortisol [µg/dL] | 12.5±2.6 | 12.5±3.7 | 12.1±4.0 | 12.4±3.1 | .98 |
| BDNF [ng/mL] | 25.8±5.6 | 31.8±8.3 | 26.4±6.9 | **21.8±9.8*** | **.01** |
| IGF-1 [µg/L] | 184±52 | 166±48 | 147±62 | 160±22 | .25 |
| Melatonin [µg/dL] | 15.8±12.7 | 26.4±13.9 | **13.5±8.6*** | 24.7±9.1 | <.01 |
| Adrenalin [µg/L] | 68.8±35.6 | 58.1±15.2 | 56.1±39.9 | 39.8±22.7 | .15 |
| Noradrenalin [µg/L] | 482±164 | 409±165 | 353±136 | **311±108^†^** | **.03** |
|  | **Post intervention** | | | |  |
| Cortisol [µg/dL] | 13.6±2.4 | 11.5±2.5 | 12.9±3.2 | 12.1±1.3 | .18 |
| BDNF [ng/mL] | 25.2±6.5 | 30.1±9.4 | 25.4±6.1 | 22.7±9.4 | .22 |
| IGF-1 [µg/L] | 172±49 | 148±40 | 157±54 | 171±60 | .63 |
| Melatonin [µg/dL] | 17.4±12.4 | 24.8±13.5 | 16.2±14.8 | 22.4±7.8 | .26 |
| Adrenalin [µg/L] | 57.4±29.9 | 59.3±34.8 | 62.1±43.8 | 30.7±20.6 | .27 |
| Noradrenalin [µg/L] | 441±173 | 289±88 | 336±177 | 272±163 | **.04** |

Values are displayed as mean ± SD and present the intravenous blood concentrations prior to and after each intervention period of BDNF (brain-derived neurotrophic factor), IGF-1 (insulin-like growth factor-1) for the groups ISO_100_, ISO_50_, CTRL_Ex_, and CTRL_NonE._. * Difference to ISO_50_ (*p* < .05); † Difference to ISO_100_ (*p* < .05).
